# Correction: Secondary dispersal driven by overland flow in drylands: Review and mechanistic model development

**DOI:** 10.1186/s40462-014-0014-5

**Published:** 2014-08-22

**Authors:** Sally E Thompson, Shmuel Assouline, Li Chen, Ana Trakhtenbrot, Tal Svoray, Gabriel Katul

**Affiliations:** Department of Civil and Environmental Engineering, UC Berkele, Berkeley, California 94720 USA; Soil, Water and Environmental Sciences, A.R.O. – Volcani Center, Bet Dagan, 50250 Israel; Division of Hydrologic Sciences, Desert Research Center, Las Vegas, NV 89119 USA; Nicholas School of the Environment, Duke University, Box 90328, Durham, North Carolina 27708 USA; Geography and Environmental Development, Ben-Gurion University of the Negev, Beer Sheva, Israel; Pratt School of Engineering, Duke University, Durham, North Carolina 27708 USA

## Correction

After publication of this work [1], we noted that we inadvertently misspelled the name of author Dr. Ana Trakhtenbrot. Here, the author list is updated to reflect the correct spelling of Dr. Trakhtenbrot’s name.
